# Automated classification of conversation valence and arousal using autonomic nervous system responses

**DOI:** 10.3389/fnins.2026.1742862

**Published:** 2026-05-20

**Authors:** Iman Chatterjee, Benjamin A. J. Miller, Joshua D. Clapp, Vesna D. Novak

**Affiliations:** 1 Department of Electrical and Computer Engineering, University of Cincinnati, Cincinnati, OH, United States; 2 Department of Psychology, University of Wyoming, Laramie, WY, United States

**Keywords:** affective computing, autonomic nervous system responses, classification, conversation, dyads, physiological computing, physiological synchrony, psychophysiology

## Abstract

**Introduction:**

The quality of dyadic cooperation or conversation can be predicted from the interacting individuals’ physiological responses, and several studies have combined physiological responses with machine learning algorithms to classify conversation states. However, most of these studies have focused on either a single physiological response or two-class classification. In this study, we used multiple autonomic nervous system responses and pattern recognition algorithms to automatically classify four dyadic conversation scenarios corresponding to four quadrants of the arousal-valence space.

**Methods:**

Heart rate, electrodermal activity, respiration, and peripheral skin temperature were measured from *N* = 51 dyads in all four scenarios, and audio/video of the dyads were simultaneously recorded. Physiological data were classified into the four scenarios using multiple different feature selection and classification algorithms. For comparison, audio/video was classified manually into the four scenarios by human raters, and dyads’ self-report questionnaire answers were classified into the four scenarios using classification algorithms as well.

**Results:**

The highest 4-class classification accuracy achieved using physiological responses was 51.5%. Conversely, human raters achieved an accuracy of 69.6% based on manual review of audio/video, and an accuracy of 79.5% was achieved using participants’ self-report questionnaire responses.

**Discussion:**

While the study is considered exploratory since many algorithms were tested, we conclude that dyadic autonomic nervous system responses can be used to classify conversation valence and arousal, but such physiological responses are not as accurate as human observers or self-report questionnaires. We finally propose potential additional analyses as well as three directions for future experiments: combining physiological responses with other data types, scenarios where participants have different perceptions of the interaction, and clinical populations.

## 1 Introduction

When two people talk to each other or work together, they coordinate their behavior on many levels: neural, perceptual, affective, and behavioral (Delaherche et al., 2012; Wheatley et al., 2012). Such interpersonal coordination can be measured using diverse sensors and used to characterize the interaction. For example, if we look only at physiological measurements, studies have shown that the quality of dyadic (i.e., two-person) cooperation can be predicted from dyads’ heart rates (HR) (Ahonen et al., 2016), electrodermal activity (EDA) (Park et al., 2022; Vanutelli et al., 2017), and electroencephalograms (Reinero et al., 2021; Szymanski et al., 2017). Similarly, the degree of interpersonal connectedness in mental health counseling, education, and conflict resolution can also be predicted from participants’ central and autonomic nervous system responses (Bar-Kalifa et al., 2019; Sun et al., 2020; Tschacher and Meier, 2020; Zheng et al., 2020). Such automated recognition of dyadic psychological states falls under the broader domain of affective computing, which uses diverse sensors and algorithms to recognize human psychological states (Aranha et al., 2021).

Since dyads’ physiological responses can be used to predict the quality of cooperation and conversation, researchers have suggested diverse uses for them. For example, group emotions could be inferred from physiological responses and visually displayed to groups of learners in order to improve collaborative learning (Järvenoja et al., 2020). Similarly, therapist-client connectedness could be visually displayed during mental health counseling sessions in order to guide the session and potentially improve its outcome (Saul et al., 2022). Such physiological analysis is particularly valuable since it may provide information not visible from self-report measures (Reinero et al., 2021) and since physiological responses may be perceived as less intrusive in sensitive situations (e.g., mental health counseling) than speech and video recordings.

Given this potential of physiological responses in dyadic settings, several studies have applied supervised machine learning algorithms to dyadic physiological responses in order to infer the interaction state – similarly to how machine learning is used with physiological responses to recognize human emotions in single-user affective computing (Aranha et al., 2021; Novak et al., 2012). However, studies involving dyadic physiology and machine learning are far less developed than studies involving single users: most of them have used classification algorithms to classify a single physiological signal (e.g., only EDA) into two (Brouwer et al., 2019; Hernandez et al., 2014; Konvalinka et al., 2014; Muszynski et al., 2018; Pan et al., 2020), three (Simar et al., 2020), or four states (Verdiere et al., 2019). Only a few studies have combined multiple physiological signals (e.g., HR and EDA) in classification (Bota et al., 2023; Chatterjee et al., 2023; Darzi and Novak, 2021; Saffaryazdi et al., 2022) or regression (Chatterjee et al., 2021, 2025), and to our knowledge no study has combined physiological signals with other types of data (e.g., speech).

Our research group is broadly interested in using physiological responses to characterize conversations, and recently conducted (to our knowledge) the only study where multiple physiological responses of two interacting participants were classified using machine learning into four classes (positive two-sided conversation, negative two-sided conversation, one-sided conversation with person A speaking, and one-sided conversation with person B speaking) (Chatterjee et al., 2023). The study achieved a four-class accuracy of 75%, but three valid concerns can be raised. First, the four-class problem was not very challenging, as one-sided conversation could likely easily be separated from two-sided conversation. Second, it is unclear whether the classification provided any information that could not have been easily obtained by an external observer or by the participants themselves. Third, the sample size was low (16 dyads), leading to concerns about whether the machine learning algorithms were adequately trained.

As the next step, the current paper presents a study where multiple autonomic nervous system responses are again classified into four classes, but with two novel elements. First, the four classes are now harder to separate as they all involve two-sided conversation and are intended to represent four quadrants of the valence-arousal axes (Bradley and Lang, 1994). Second, the classification accuracy is compared to the accuracy achieved by human raters watching videos of the conversation, providing an estimate of how well similar information could be obtained from other data – as recommended by review studies of methods in the field (Brouwer et al., 2015). In addition to these novel elements, the sample size of *N* = 51 dyads is larger than (to our knowledge) all previous studies involving classification of dyadic physiological responses. Combining autonomic nervous system responses with other sources of data in machine learning, while relevant in the long term, was considered beyond the scope of the current study.

## 2 Materials and methods

**Figure 1:**
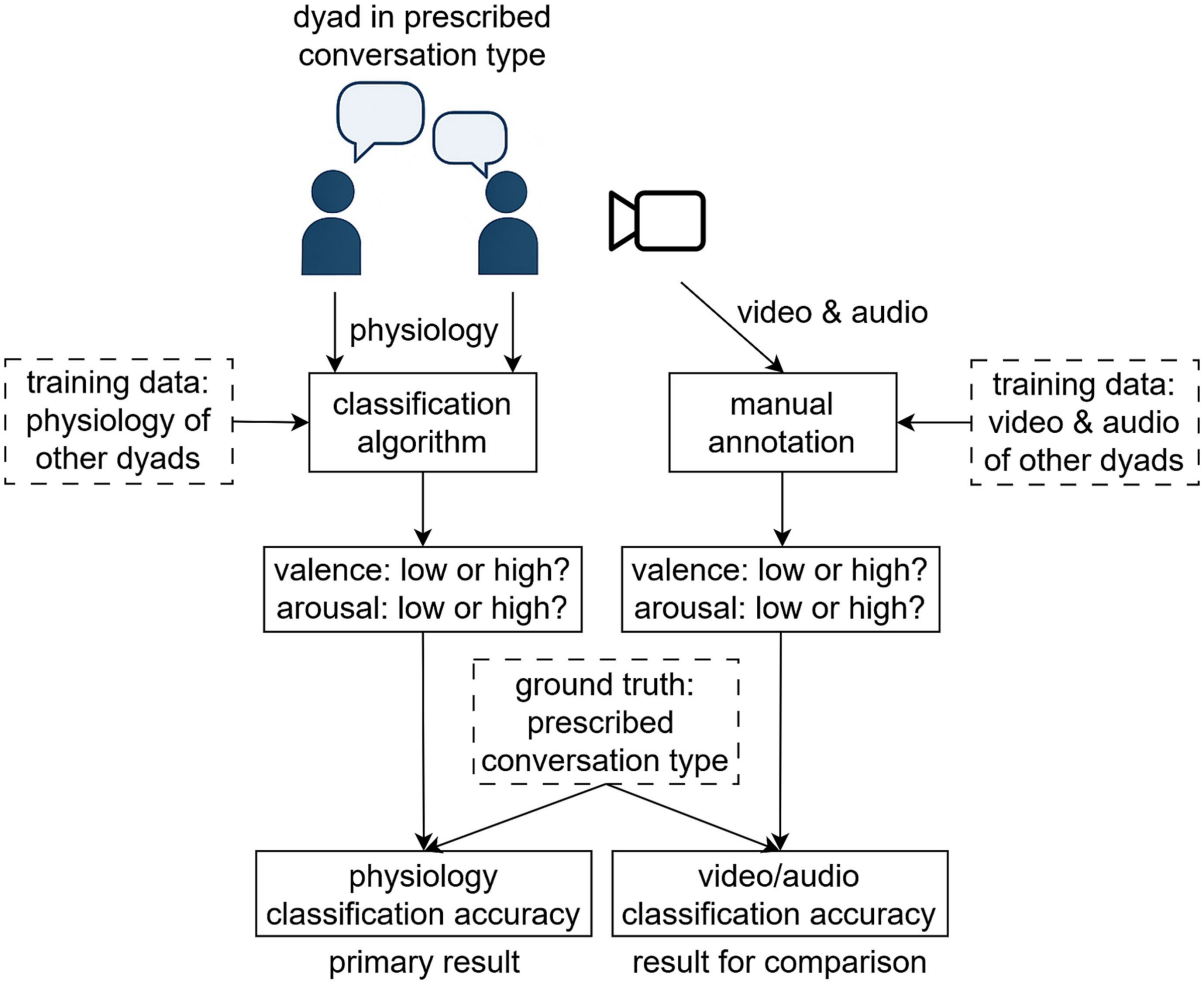
The overall study concept. The study aims to classify physiological responses of conversing dyads into one of four classes (negative/positive valence, low/high arousal) using supervised machine learning trained on data from other dyads. Manual annotation of audio/video data is also done for comparison, and the prescribed conversation type is used as the ground truth.

The overall goal of our study was to classify dyads’ autonomic nervous system responses into four classes corresponding to four conversation scenarios. To do this, multiple dyads (section 2.4) went through a study protocol where they engaged in the four conversation scenarios (section 2.1). Physiological, self-report, and audio/video data were collected (section 2.2). We used the self-report and audio/video data to verify that the conversation types were successfully induced and to evaluate how well a human observer can classify audio/video data into the four classes (section 2.3). Physiological features were then extracted from the raw signals (section 2.5) and input into multiple feature selection and classification algorithms (section 2.6). The overall study concept is illustrated in Figure 1.

### 2.1 Study protocol

The study was approved by the Institutional Review Board of the University of Cincinnati (study 2021-1,107). As mentioned, our goal was to classify dyadic physiological data into four classes corresponding to four quadrants of the arousal-valence space (Bradley and Lang, 1994). Such classification is a classic challenge in single-user affective computing (Novak et al., 2012) and requires data with known arousal/valence labels. These data can be obtained in two ways. First, it is possible to observe participants and use self-report or observer ratings to assign labels to different time intervals. Second, an experimental manipulation can be used to try to induce desired psychological states in participants: for example, by asking participants to act out different emotions (Picard et al., 2001), asking them to recall positive or negative memories (Chanel et al., 2009), asking them to self-select and listen to songs that would put them into desired arousal-valence quadrants (Kim and André, 2008), or by having them watch emotionally charged videos (Arroyo-Palacios and Romano, 2010). Both approaches have weaknesses: Observing participants during naturalistic interaction may obtain an unbalanced dataset with more positive and neutral emotions than negative emotions, as seen in our previous work (Chatterjee et al., 2021, 2025; Darzi and Novak, 2021). On the other hand, experimental manipulations may not be successful in all participants, as some may not experience the desired emotions or may feel them more/less strongly (Chanel et al., 2009; Picard et al., 2001). Based on prior positive experiences with an experimental dyadic manipulation (Chatterjee et al., 2023), we chose the second approach.

Participants were recruited from the University of Cincinnati student community as self-selected (already acquainted) dyads, and each dyad took part in a single session that was 75–90 min long. Only acquainted dyads were included since our previous studies found that they were easier to work with and more likely to follow study instructions than unacquainted dyads (Chatterjee et al., 2025). Data were collected from March to November 2023.

Each session began with an explanation of the study. Participants sat opposite one another, about 1.5 m apart. They first provided written responses to three self-report trait questionnaires (described later). Guided by the researcher, participants then self-attached the physiological sensors, and signal quality was checked. The participants provided a set of noncontroversial topics that they mildly agreed on, emphatically agreed on, mildly disagreed on, and emphatically disagreed on. Generally, each dyad provided one or two topics for each of these four possibilities; the topics are described further below. This mimics earlier nondyadic work where participants were asked to self-select songs that would put them into desired states (Kim and André, 2008).

After obtaining the list of topics, baseline physiological measurements were recorded for four minutes. During this baseline, participants were told to stay silent with eyes open and a neutral facial expression. After this, the dyads went through four 4-min conversation scenarios intended to induce four emotional states corresponding to quadrants of the arousal-valence space:

- Positive-valence, high-arousal (PV-HA) conversation: Participants were told to discuss a topic they agreed on and to emphasize their agreement/positivity throughout the conversation – i.e., emphatically agree with the other person’s statements and appear cheerful/enthusiastic. They were instructed to choose a topic that had previously produced passionate conversations. While topics varied greatly between dyads, common ones included sports teams, food, or activities that both participants strongly liked.
- Positive-valence, low-arousal (PV-LA) conversation: Participants were told to discuss a topic that they mildly agreed upon. They were told to speak casually while generally agreeing with whatever was being said by the other person and staying positive. They were instructed to choose a topic that had previously caused low-energy interactions. Common topics included, e.g., weather or upcoming nonstressful school activities.
- Negative-valence, high-arousal (NV-HA) conversation: Participants were told to discuss a topic they disagreed on and to emphasize their disagreement throughout the conversation – i.e., vehemently disagree with things that the other person said and appear stubborn or even hostile. They were told to choose a topic on which they had had animated arguments before. Expressing strong opinions was allowed. Common topics included, e.g., sports team rivalries or food that one participant liked and the other strongly disliked.
- Negative-valence, low-arousal (NV-LA) conversation: Participants were told to discuss a topic that they mildly disagreed on. They were asked to speak casually while generally disagreeing with whatever was being said by the other person. They were instructed to choose a topic about which they had previously had casual disagreement. Common topics included, e.g., food that one participant mildly liked and the other mildly disliked.

Dyads could switch topics within a scenario as long as the basic nature of the scenario remained unchanged – i.e. in PV-HA, participants could agree and stay positive about multiple topics but should not begin disagreeing. While we acknowledge that many human conversations fall in a more neutral space, we intentionally tried to induce four relatively separable scenarios as done in classic studies of physiology classification in single-user affective computing (Picard et al., 2001).

The four scenarios were done in random order. After each scenario, participants completed the Self-Assessment Manikin (SAM) (Bradley and Lang, 1994) to report conversation valence and arousal as well as the Interpersonal Interaction Questionnaire (IIQ) (Goršič et al., 2019) to report the amount of conversation, its balance, and its valence. After the fourth scenario, a second 4-min baseline measurement was taken. Participants then removed the sensors and were given a $15 gift card.

### 2.2 Measurements

Physiological signals were gathered from both participants using a pair of g.USBamp amplifiers (g.tec Medical Engineering GmbH, Austria) and associated sensors (all manufactured by g.tec). For each participant, four electrodes on the torso were used to measure the electrocardiogram (ECG). A thermistor-based Respiration Airflow sensor was positioned under the nostrils to record respiration. EDA was collected from the distal phalanges of the forefinger and middle finger of the nondominant hand using a g.GSRsensor2 sensor. A g.Temp sensor on the distal phalanx of the little finger recorded peripheral skin temperature. Signals were sampled at 600 Hz and analog 60-Hz notch filters were applied. A 0.1-Hz highpass analog filter was applied to the ECG while the other signals were filtered using analog 30-Hz lowpass filters.

In addition to physiological data, three self-report trait questionnaires were filled out. The Questionnaire of Cognitive and Affective Empathy (Reniers et al., 2011) measured cognitive and affective empathy, the Brief Fear of Negative Evaluation Scale (Leary, 1983) measured social anxiety, and the Center for Epidemiologic Studies Depression Scale (Leary, 1983) measured depression. These four traits were shown to influence dyadic physiological responses in prior work (McKillop and Connell, 2018; Sachs et al., 2020; Steiger et al., 2019).

Finally, videos of both participants were collected using two consumer-grade webcams placed on the table between the participants, with one webcam pointed at each participant’s face. Both participants’ speech was recorded using a Yeti X microphone (Blue Microphones, USA).

### 2.3 Manual analysis of nonphysiological data

Audio/video and SAM/IIQ data were analyzed to verify that the desired scenarios were successfully induced and to determine how well human observers could classify the assigned scenarios. First, SAM and IIQ responses were examined by author IC for all dyads in all scenarios. Cases in which self-report responses did not appear to match those of the assigned scenario (e.g., participants reporting positive valence in a negative-valence scenario) and cases where the experimenter noted potential noncompliance during data collection were flagged for further evaluation. Video recordings from potentially non-compliant dyads were reviewed by authors IC and VDN, with dyads excluded from all further analysis if both authors felt that the dyad had failed to adhere to experimental instructions in at least one of the four scenarios (PV-HA, PV-LA, NV-HA, NV-LA). This was done since noncompliance is known to be relatively high in dyadic conversation studies (Roberts et al., 2007) and we felt that it would not make sense to include noncompliant dyads in analysis.

Next, audio/video recordings from valid dyads were separated into files corresponding to each individual 4-min scenario. Segment files were given a random name and randomly assigned to one of four external raters (3 predoctoral psychology students and one advanced psychology undergraduate, all supervised by author JDC). These raters were given a description of the four scenarios and viewed three examples of each scenario from three pilot dyads (not included in final analysis), then rated their assigned segments – i.e., classified each segment as one of the four scenarios. The separation and randomization were intended to (a) reduce potential impact of systematic differences in rater accuracy and (b) minimize the potential for raters to rate a given segment based on previous ratings of that same dyad (e.g., knowing that each dyad experiences all scenarios and rating a dyad’s fourth segment via process of elimination given the dyad’s previous three ratings). Finally, prior to rating, 25% of segments were randomly selected to be rated by two raters to provide an estimate of interrater reliability. This process overall allowed us to estimate how well a human can identify the conversation scenario based on audio/video data and evaluate how the differences between individual raters may influence their classification accuracy.

### 2.4 Participants

Fifty-eight dyads completed data collection. Data from two dyads were discarded due to excessive noise in physiological signals, and data from an additional five dyads were discarded due to failure to comply with experimental instructions in at least one scenario (primarily failure to exhibit negative valence in NV-HA and/or NV-LA). These seven dyads were excluded from any further analyses, which were thus conducted with 51 valid dyads.

The ages of these participants (ranging from 17 to 29 years) were 19.8 ± 2.1 years (mean ± SD). Nineteen dyads consisted of two women, 15 of two men, 14 of one woman and one man, two of one woman and one nonbinary individual, and one of two nonbinary individuals. Across the 51 dyads, trait questionnaire scores were 39.2 ± 9.7 for social anxiety (theoretical possible range 19–56), 25.9 ± 10.7 for depression (possible range 9–52), 57.9 ± 7.2 for cognitive empathy (range 43–76), and 35.3 ± 5.5 for affective empathy (range 20–45).

### 2.5 Physiological feature extraction

Each dyad’s physiological signals were split into six segments: a 4-min initial baseline, the four 4-min conversation scenarios, and the 4-min final baseline. Any data that did not lie in these segments were removed.

For each of these segments, we applied Butterworth digital 5-Hz fourth-order lowpass filters to the EDA, respiration, and skin temperature inputs. MATLAB peak detection logic was used to identify ECG peaks corresponding to R-waves, which were manually examined and corrected if needed to ensure that they truly represented R-waves. If a peak could not be identified due to noise, peaks were interpolated midway between two valid R-waves. A similar method was used to identify peaks that represented individual breaths in the respiration signal. A peak detection algorithm from our previous study (Chatterjee et al., 2023) was applied to the EDA signal to identify individual quick surges in EDA known as skin conductance responses (SCRs).

Once data had been preprocessed, features were extracted from the six baseline and conversation segments. These features were categorized either as individual features (computed from physiological data of a single participant) or synchrony features (computed from physiological data of both participants). The individual features were as follows:

- ECG: Mean HR, minimum HR, maximum HR, three time-domain measures of HR variability (standard deviation (SD) of interbeat intervals, root-mean-square value of consecutive differences between these intervals, percentage of consecutive interbeat intervals with a difference greater than 50 ms), and three frequency-domain measures of HR variability (power in low-frequency band, power in high-frequency band, the ratio of the two). These features are common in affective computing studies (Task Force of the European Society of Cardiology and the North American Society of Pacing and Electrophysiology, 1996).
- EDA: Mean EDA, difference between initial and final EDA values, number of SCRs, mean SCR amplitude, SD of SCR amplitudes.
- Respiration: mean respiration rate and SD of respiratory periods.
- Peripheral skin temperature: mean temperature, difference between initial and final temperature values.

The synchrony features reflected the degree of physiological synchrony between the dyad members. They were computed using instantaneous HR and instantaneous respiration rate [computed as functions of time from raw measurements of ECG and respiration (Darzi and Novak, 2021)] as well as filtered skin temperature and EDA signals. Synchrony features were the same for all four signal types and were as follows:

- Dynamic time warping distance, calculated via the dynamic programming-based method proposed by Muszynski et al. (2018). This approach attempts to measure the similarity between two signals and is resistant to time delays in individual signals (Hernandez et al., 2014).
- Nonlinear interdependence, also calculated using a technique proposed by Muszynski et al. (2018). This feature quantifies the geometric similarity between state-space trajectories of two dynamical systems. Trajectories representative of shape distribution distance were described using time-delay embeddings (Muszynski et al., 2018).
- Coherence, which was calculated using a method from our previous study (Darzi and Novak, 2021) that checked if the oscillations of the two signals lay in the same single frequency band or whether they extended to multiple frequency bands. The feature was calculated for multiple frequency ranges for the different signals (Darzi and Novak, 2021).
- Cross-correlation, computed using the same approach as our previous study (Darzi and Novak, 2021). This feature simply measured the Pearson correlation between the signals of the two participants of a dyad (Hernandez et al., 2014).

MATLAB code that computes synchrony features is available on Zenodo (Chatterjee et al., 2022). For each dyad and each feature, normalized feature values were obtained for the four conversation segments by subtracting the values from the first baseline segment. Such normalization is common in affective computing since it is believed to reduce intersubject variability (Aranha et al., 2021; Novak et al., 2012).

Finally, after normalization, the normalized individual features of both participants in the dyad were combined: they were originally calculated for each participant, but since both participants have the same role in the conversation, there is no conceptual difference between a feature from one participant and the same feature from the other participant. Therefore, for each individual feature, we calculated:

- the mean feature value of both participants: for example, mean(mean heart rate) = (mean heart rate of participant on left + mean heart rate of participant on right)/2
- the absolute difference of the feature values of both participants: for example, absdiff(mean heart rate) = abs(mean heart rate of participant on left – mean heart rate of participant on right)

This approach is inspired by a previous study that used a similar approach (Hernandez et al., 2014), though there is no standard way to combine individual features from two participants who have the same role; we return to this in the Discussion. This was not done for synchrony features, which are already calculated from data of both participants.

In the end, 52 physiological features were available for classification: 36 individual features (9 ECG + 5 EDA + 2 respiration + 2 peripheral skin temperature = 18, multiplied by 2 for the mean + absolute difference of both participants) and 16 synchrony features (4 feature types x 4 signals).

### 2.6 Classification of physiological features

#### 2.6.1 Direct, serial, and parallel classification

As mentioned, the primary goal of our study is to distinguish among four conversation scenarios based on physiological data. This was done by applying classifiers to normalized physiological feature values obtained from all conversation segments. However, as there were more than two classes, four classification approaches were used:

- Direct 4-class classification: A 4-class classifier was used to directly classify the conversation segments as one of the four conversation scenarios.
- Serial binary classification, arousal first: In the first stage, features were classified as high-arousal or low-arousal conversation with a binary classifier. In the second stage, data classified as high arousal were further classified as PV-HA or NV-HA while data classified as low arousal were classified as PV-LA or NV-LA.
- Serial binary classification, valence first: Similar to the previous case, but features were first classified as positive-valence or negative-valence.
- Parallel binary classification: One binary classifier was used to classify features as high vs. low arousal while another binary classifier classified them as positive vs. negative valence. The outcomes of these were then combined to classify the features into one of the four scenarios.

Serial and parallel classification were *a priori* expected to outperform direct 4-class classification since each individual classifier has the same number of training data points but must deal with a simpler binary problem.

#### 2.6.2 Classifiers and feature selection algorithms

In all four classification approaches, six supervised classifiers were used: k-nearest neighbors (kNN), linear discriminant analysis, support vector machine, naïve Bayes, gradient boosting, and random forest. Standard MATLAB 2021b (Mathworks, USA) functions were used to implement these: *fitcknn* (kNN)*, classify* (linear discriminant analysis), *fitcsvm* (support vector machine), *fitcnb* (*naïve Bayes*)*, fitcensemble* (gradient boosting), and *treebagger* (random forest). Hyperparameter optimization techniques available in these functions were used to obtain optimal values for parameters such as number of neighbors for kNN. For full disclosure, we note that other classifiers such as gentle adaptive boosting were also tested with the data but are not reported in detail since they yielded relatively low accuracies.

To avoid overfitting, we used feature selection to identify the most representative feature subsets before classification. For this, we used five standard techniques in MATLAB 2021b and Python 3.1.2: bidirectional stepwise feature selection (using MATLAB *stepwisefit* function), maximum relevance minimum redundancy test for univariate feature ranking (using MATLAB *fscmrmr* function), relief-based feature selection (using MATLAB *relieff* function), chi-square function-based selection (using MATLAB *fscchi2* function), and mutual information-based feature selection (using *mutual_info_classif* in Python).

Since there were 5 feature selection algorithms and 6 classification algorithms, the direct 4-class classification approach involved 30 (5×6) tested algorithm combinations. Different combinations of algorithms were tested for the different stages of the serial and parallel approaches to obtain the best combination for each stage. Therefore, the parallel approach involved 900 (30×30) tested algorithm combinations, and the serial approaches involved 27,000 combinations.

#### 2.6.3 Primary classifier evaluation

Classification approaches were evaluated using leave-dyad-out crossvalidation: feature selection and classification algorithms were trained on data from all but one dyad and tested on the remaining single dyad. This process was repeated as many times as there were dyads, with each dyad used as the test dyad once. Mean classification accuracy across all test dyads was computed as the overall outcome similarly to our previous work (Chatterjee et al., 2023). The mean accuracies of 4-class, serial, and parallel binary classification based on physiological features served as the results of our primary analysis. *A priori*, we expected that the accuracy would be lower than the 75% seen in our prior work (Chatterjee et al., 2023) since that study involved four more easily separable scenarios. However, we also hoped that the accuracy would be similar to that achieved by external raters of video recordings (section 2.3).

Once the most effective feature selection and classification algorithms had been determined via crossvalidation for each of the four approaches (direct 4-class, parallel, and two serial approaches), permutation tests were used to compare the results of the four approaches against random chance. Within each dyad, the scenario labels were randomly shuffled and the same feature selection and classification algorithm was retrained on the shuffled dataset; this was done 1,000 times, and the accuracies of the four approaches were compared to the mean accuracies of the same approaches done on permuted data. Then, the accuracies of the four approaches were compared to each other using a one-way repeated-measures analysis of variance on ranks (RMANOVA). This RMANOVA was done on 4×51 data points: 4 approaches x 51 accuracies from 51 dyads. RMANOVA on ranks was considered appropriate since accuracy within a dyad has only 5 possible values (0, 0.25, 0.5, 0.75, 1) that are not normally distributed. RMANOVA was followed by post-hoc Tukey tests to compare individual approaches.

#### 2.6.4 Secondary analyses

In addition to the primary analysis, we also performed four secondary analyses. These secondary analyses were based on recommendations from review papers in the field of physiology classification, which suggest that the contributions of different features should be evaluated and the most important features should be identified (Brouwer et al., 2015). They were:

1. In the first secondary analysis, the same classifiers were created using only individual physiological features as inputs – i.e., synchrony features were excluded from this analysis. We *a priori* expected that removing synchrony features would reduce accuracy since it would reduce insights into interpersonal dynamics. However, synchrony features are not as well established in affective computing as individual physiological features, and they require appropriate temporal synchronization between both participants’ sensors. Thus, they should be only included in practical applications if they indeed provide information not obtainable from individual features, and we wished to verify this.
2. In the second analysis, the participants’ self-reported personality traits (social anxiety, affective empathy, cognitive empathy, depression) were added to the input dataset. As with individual physiological features, these were first converted from individual participants’ values to the mean and absolute difference of each trait within the dyad. *A priori*, we expected that this would not improve accuracy since our recent classification study found that adding personality traits did not affect accuracy (Chatterjee et al., 2023). However, another less closely related study did find accuracy improvements when including personality traits (Chatterjee et al., 2021), so we felt that this should be evaluated in this study as well. As personality traits are measured with long questionnaires (taking 10–15 min per session) that are potentially intrusive, they should only be measured if they provide additional information for classification.
3. In the third analysis, we identified the five most relevant features for the algorithms yielding the highest accuracies in our primary analysis. This was done by first identifying the best feature selection algorithm in the primary analysis, then applying it to the full dataset of 51 dyads and listing the 5 features considered most important by that feature selection algorithm.
4. In the final analysis, the same classifiers were first created using only the dyads’ SAM and IIQ values (again converted to mean and absolute difference of each item within the dyad), but without physiological or personality trait data. The classifiers were then created using both physiological data and SAM and IIQ values (but no personality trait data). A one-way RMANOVA followed by post-hoc Tukey tests was applied to compare three approaches: the most accurate physiology-based classification approach, the most accurate approach based on only SAM and IIQ, and the most accurate approach using both physiology and SAM and IIQ values (3×51 data points). This allowed us to evaluate whether physiological data can provide better information than simply asking participants how they feel as well as whether they can complement self-report data.

## 3 Results

The 51 valid dyads’ SAM and IIQ responses are shown in Table 1. Physiological features computed for all dyads and intervals are available on Zenodo (see Data Availability Statement).

**Table 1:** Self-Assessment Manikin and Interpersonal Interaction Questionnaire values for all four conversation scenarios.

Scenario	Self-Assessment Manikin				Interpersonal Interaction Questionnaire			
	Valence		Arousal		Balance		Valence	
	Overall	Abs. diff.	Overall	Abs. diff.	Overall	Abs. diff.	Overall	Abs. diff.
Positive valence High arousal	8.4 ± 0.8	0.6 ± 0.8	7.0 ± 1.7	1.4 ± 1.5	3.7 ± 1.1	1.1 ± 0.9	3.4 ± 0.9	0.7 ± 0.7
Positive valence Low arousal	7.0 ± 1.2	1.1 ± 1.1	4.6 ± 1.7	1.8 ± 1.2	3.8 ± 1.2	1.1 ± 0.9	3.4 ± 0.9	0.6 ± 0.7
Negative valence Low arousal	4.6 ± 1.7	1.7 ± 1.7	5.2 ± 1.7	1.6 ± 1.4	3.9 ± 1.2	1.2 ± 1.1	1.4 ± 0.6	0.3 ± 0.5
Negative valence High arousal	4.4 ± 2.4	2.4 ± 1.9	7.4 ± 1.5	1.1 ± 1.2	3.9 ± 1.2	1.2 ± 1.0	2.0 ± 0.7	0.7 ± 0.6

“Overall” columns represent that questionnaire item across all 102 participants while “abs. diff.” columns represent the absolute difference of that item between the two participants within a dyad, across all 51 dyads. Results given as mean ± standard deviation. Self-Assessment Manikin values have a range of 1–9, with 9 denoting high valence/arousal. Interpersonal Interaction Questionnaire values represent items 3 (“How balanced was the conversation?”) and 6 (“How would you rate the overall conversation?”) and have a range of 1–5, with 5 indicating balanced or positive-valence conversation.

Human raters achieved an accuracy of 69.6% across all 204 conversation segments based on review of audio/video records. Agreement between independent raters on randomly selected reliability segments was marginally higher at 76.5%. While a full confusion matrix is not provided, raters were relatively good at classifying valence compared to arousal – only 3 of 204 segments were incorrectly rated with regard to valence.

### 3.1 Primary analysis

Table 2 shows results for the four physiological feature classification approaches described earlier. The table also includes the specific feature selection and classification algorithms used in each approach. The highest accuracy was 51.5%, obtained using the serial binary classification approach where valence was classified first. In this approach, the first-stage accuracy of valence classification was 67.2% while second-stage arousal classification accuracies were 78.9% (high-valence cases) and 74.2% (low-valence cases); the full two-stage confusion matrix is given in Table 3. Permutation tests showed that all four approaches outperformed random chance (*p* < 0.05 for all four), but the RMANOVA did not find a significant difference between the four approaches (*p* = 0.08).

**Table 2:** Classification accuracies in the primary analysis, presented as mean ± standard deviation across the 51 dyads.

Approach	Classifier + feature selection		Accuracy (overall)
	1st stage	2nd stage	
Serial, valence first	gradient boosting + relief-based	gradient boosting + relief-based gradient boosting + relief-based	51.5% ± 20.9%
Serial, arousal first	random forest + stepwise	random forest + MRMR random forest + MRMR	41.2% ± 25.4%
Parallel	random forest + stepwise (for arousal)	gradient boosting + MRMR (for valence)	42.2% ± 24.7%
Direct 4-class	random forest + chi-square	None	41.7% ± 21.7%

MRMR, maximum relevance minimum redundance-based selection.

**Table 3:** The overall confusion matrix for the serial valence-first approach in the primary analysis.

Actual class	Predicted class			
	Positive valence, high arousal	Positive valence, low arousal	Negative valence, high arousal	Negative valence, low arousal
Positive valence, high arousal	26	7	18	0
Positive valence, low arousal	8	30	13	0
Negative valence, high arousal	20	0	18	13
Negative valence, low arousal	16	0	4	31

Numbers in cells represent the number of samples.

More information about specific algorithm parameters and selected features in the four approaches is available via the Data Availability Statement. While a detailed comparison of different classifiers and feature selection methods in each approach is not presented, the highest accuracy was consistently achieved using either gradient boosting or random forests as classifiers (Table 2). Replacing gradient boosting with random forests or vice versa generally led to only slightly lower accuracy; on the other hand, using other classifiers in the first stage often led to noticeably lower accuracy. This was not the case in the second stage, where other classifiers also tended to achieve similar accuracy to random forests and gradient boosting.

### 3.2 Secondary analyses

For our first secondary analysis, synchrony features were omitted from the dataset. Table 4 shows results of this analysis for all four classification approaches. The highest accuracy was 43.1% using the serial binary classification approach where valence was classified first. Permutation tests found that all four approaches still outperformed random chance (*p* < 0.05 for all), but RMANOVA did not find a significant difference between the four approaches (*p* = 0.85).

**Table 4:** Classification accuracies in the secondary analysis where synchrony features were removed.

Approach	Classifier + feature selection		Accuracy (overall)
	1st stage	2nd stage	
Serial, valence first	gradient boosting + relief-based	gradient boosting + relief-based LDA + relief-based	43.1% ± 26.0%
Serial, arousal first	naïve Bayes + stepwise	LDA + MRMR random forest + MRMR	37.3% ± 17.6%
Parallel	LDA + stepwise (for arousal)	random forest + MRMR (for valence)	41.7% ± 26.3%
Direct 4-class	random forest + chi-square	none	38.2% ± 18.3%

MRMR, maximum relevance minimum redundance-based selection; LDA, linear discriminant analysis.

For our second secondary analysis, we added personality features to the dataset to estimate the impact of these additional features. Accuracies obtained in this case were never higher than those observed in primary analyses and are thus not shown in detail.

In the third secondary analysis, we identified the most important features for each of our classifiers. These are listed in Table 5.

**Table 5:** The five most important features for different classifiers in the primary analysis.

Approach	Stage	Feature rank	Feature
Serial, valence first	First (valence)	1	absdiff (mean temperature)
		2	absdiff (difference between initial & final temperature)
		3	mean (minimum heart rate)
		4	absdiff (mean heart rate)
		5	mean (mean temperature)
	Second (arousal for high valence)	1	absdiff (mean temperature)
		2	cross-correlation of heart rate
		3	absdiff (mean respiration rate)
		4	mean (maximum heart rate)
		5	coherence of heart rate
	Second (arousal for low valence)	1	mean (maximum heart rate)
		2	absdiff (high-frequency heart rate variability)
		3	mean (mean skin conductance response amplitude)
		4	absdiff (difference between initial & final EDA values)
		5	mean (high-frequency heart rate variability)
Serial, arousal first	First (arousal)	1	absdiff (number of skin conductance responses)
		2	coherence of heart rate
		3	mean (mean heart rate)
		4	mean (low-frequency heart rate variability)
		5	coherence of respiration rate
	Second (valence for high arousal)	1	cross-correlation of heart rate
		2	absdiff (minimum heart rate)
		3	absdiff (standard deviation of respiratory periods)
		4	mean (mean respiration rate)
		5	absdiff (mean electrodermal activity)
	Second (valence for low arousal)	1	mean (mean temperature)
		2	cross-correlation of heart rate
		3	mean (mean skin conductance response amplitude)
		4	coherence of heart rate
		5	absdiff (low-frequency heart rate variability)
Parallel	Arousal	1	cross-correlation of heart rate
		2	mean (maximum heart rate)
		3	mean (low-frequency heart rate variability)
		4	mean (mean temperature)
		5	mean (high-frequency heart rate variability)
	Valence	1	dynamic time warping distance of respiration rate
		2	cross-correlation of heart rate
		3	absdiff (mean temperature)
		4	cross-correlation of respiration rate
		5	mean (mean electrodermal activity)
Direct 4-class	Single stage	1	absdiff (standard deviation of NN intervals)
		2	cross-correlation of heart rate
		3	mean (maximum heart rate)
		4	mean (standard deviation of NN intervals)
		5	mean (mean heart rate)

There are four classification approaches, and some have multiple stages, so those classifiers are presented separately. Mean() and absdiff() represent features obtained separately from individual participants and then either averaged or subtracted. EDA, electrodermal activity.

In the final secondary analysis, classification was first done based on only SAM and IIQ data, no physiology. In this case, the highest accuracy was 79.5% ± 21.2%, obtained using the direct 4-class approach. Classification was then done based on physiology together with SAM and IIQ data. In this case, the highest accuracy was 82.5% ± 20.4%, again obtained using the direct 4-class approach. As with human raters, these approaches were relatively good at classifying valence compared to arousal, with about 4% of segments incorrectly rated compared to valence. Finally, RMANOVA was used to compare these approaches to the most accurate physiology-based approach from the primary analysis (serial valence-first, with 51.5% accuracy). It found that the approaches were different (*p* < 0.001); post-hoc tests found that both approaches involving SAM and IIQ data resulted in higher accuracy that the physiology-based approach (*p* < 0.001 for both), but that the approach with only SAM and IIQ data was not significantly different from the approach with physiology, SAM, and IIQ data (*p* = 0.21).

## 4 Discussion

The highest 4-class classification accuracy was 51.5%, which is much higher than the 25% chance value for balanced 4-class classification, higher than the 35% accuracy obtained using only electroencephalography in another 4-class classification study (Verdiere et al., 2019), and similar to 4-class leave-subject-out classification accuracies obtained with traditional features and machine learning algorithms in single-user affective computing (Novak et al., 2012). Accuracy decreased to 43.1% when synchrony features were removed in the secondary analysis. Together, these two results indicate that classification algorithms can be applied to autonomic nervous system responses to classify the state of a conversation and that physiological synchrony provides additional information beyond what can be obtained from the two participants’ individual physiological features.

Less encouragingly, the accuracy of 51.5% was lower than the 75.0% achieved in our previous study where similar 4-class classification was performed with more easily separable classes (Chatterjee et al., 2023). It was also lower than the accuracy achieved by the human raters (69.6%) or the accuracy obtained from the dyads’ own self-report data (79.5%). The imperfect accuracies achieved by observers and self-report data do imply that the four scenarios are not perfectly separable despite removal of dyads who did not follow study instructions. For example, the 79.5% obtained for classification using self-report data was likely partially because different participants experience different internal states in the four scenarios (e.g., some may get strongly upset in the NV-HA scenario while others may experience only mild annoyance) and partially because participants sometimes do not reliably self-report psychological states, as seen in our prior dyadic work with valence and arousal (Chatterjee et al., 2023, 2025) and frequently reported in others’ work (Brouwer et al., 2015). Since different participants experience somewhat different psychological states within the same scenario, their physiological responses are also different and 100% accuracy should not be expected from physiological classification.

However, since physiological responses yielded lower accuracies than external observation or self-report data, and since adding physiological data to self-report data only increased accuracy by 3%, the physiological responses do not appear to provide additional value over simply observing each person’s behavior or asking them how they feel. Thus, while the results of the study are scientifically valid, they suggest that classification of dyadic autonomic nervous system responses has limited practical utility both because it does not outperform other data types (Brouwer et al., 2015) and because user satisfaction with physiology classification is directly correlated with classification accuracy (Novak et al., 2024).

### 4.1 Comparison of approaches in physiology-based classification

Aside from comparing the accuracy of physiology-based classification to the accuracy of human raters and self-report data, it is also possible to compare different approaches within physiology-based classification. First, Table 5 shows that HR features tend to appear most commonly among the most important features for classification, but all four physiological signals are represented in the table. Inspection of the full lists of selected features (see Data Availability Statement) shows that HR features are also relatively common in the full selected feature lists. This may simply be because more individual features were extracted from HR than from the other three signals; simultaneously, HR is considered to be perhaps the most information-rich autonomic nervous system response in single-user affective computing (Aranha et al., 2021), so it is not surprising that it also appears often in our classifiers. Nonetheless, features from the other three signals do appear in most classifiers, and thus none of the four signals can clearly be excluded from future studies. This result is similar to those in our previous dyadic studies, where multiple autonomic nervous system responses tended to contribute to classification (Chatterjee et al., 2023) or regression (Chatterjee et al., 2025).

The highest accuracy decreased from 51.5 to 43.1% when synchrony features were removed, indicating that synchrony provides additional information. However, the most important features in Table 5 are predominantly individual rather than synchrony features, indicating that synchrony features are less important. We believe this is likely because, as noted in the literature (Palumbo et al., 2017), synchrony effects are often small and context-dependent, often contributing to a model in a distributed manner that is dependent on the interaction. Thus, their utility arises from how they interact with individual physiological features, and they tend to be selected only once individual physiological features have already been included in the model. The full list of features selected in the four classification approaches (see Data Availability Statement) also does show that several synchrony features are used in each approach even if they do not appear among the most important features in Table 5. Among the individual features, both the mean feature value of both participants and the absolute difference of the feature values of both participants appear frequently among selected features, suggesting that the difference between the two participants is important even though both participants have essentially the same role in the conversation.

As seen in Table 2, the highest accuracy value was obtained for serial valence-based classification, which makes sense to us: valence was found to be easier to classify than arousal by both human observers and classification of self-report data (last secondary analysis), so it may make sense to also start classification of physiological data with the easier valence problem. However, this cannot be claimed with certainty since RMANOVA did not find a significant difference between the four approaches (*p* = 0.08) and the other three approaches all achieved about 42% accuracy.

Finally, personality traits did not improve classification accuracy when added to the physiological data, indicating that they were not useful for this problem. The same result (personality not improving accuracy) was observed in our previous classification study (Chatterjee et al., 2023) and may be because the four scenarios are relatively rigid (with chosen topics and behavior instructions), so personality has a limited impact. Our previous regression study did find benefits of including personality traits (Chatterjee et al., 2021), but that may have been because that study was less rigid, with freeflowing conversation and fewer instructions. Similarly, single-user physiology classification studies have found benefits of personality traits in less rigid scenarios (Darzi et al., 2019), supporting this explanation.

### 4.2 Future research directions

Since the utility of dyadic physiological responses for characterization of conversations is unclear based on our results, we recommend several directions for future research. They can be divided into additional analytical work and different experimental approaches.

#### 4.2.1 Potential additional analyses

First, we acknowledge that many possible algorithms were tested. We had 5 feature selection algorithms and 6 classification algorithms, so 30 combinations were tested for direct 4-class classification alone. For the parallel approach, which involves two independent subproblems, a total of 900 combinations were tested, and in the serial approaches, the number rises to 27,000. Results of individual algorithms were of course correlated: for example, in the serial approaches, an inaccurate algorithm in the first stage leads to poor overall performance regardless of accuracy in the second stage; as another example, gradient boosting and random forests tended to lead to higher accuracy (section 3.1). Nonetheless, given the large number of tested algorithms, the overall results may not be robust or stable, and our study can only be considered exploratory. Follow-up validation is needed to demonstrate that the current accuracies are reliable, and could involve, e.g., testing the same methods on a different dataset obtained using the same protocol. However, considering that the current accuracy (51.5%) is relatively low compared to that of self-report or observer ratings, such stability analysis may not be worthwhile until the accuracy can be improved further.

Second, we combined individual features from the two participants by taking the mean and absolute difference of the two participants’ values for the same feature. This is not a well-established approach, as many dyadic studies only use synchrony features with no individual features (Bota et al., 2023; Brouwer et al., 2019; Muszynski et al., 2018; Pan et al., 2020). Our own previous studies (Chatterjee et al., 2021, 2023, 2025) and some other studies (Konvalinka et al., 2014) simply passed the features to the classification algorithms as, e.g., “mean heart rate of participant on left” and “mean heart rate of participant on right”, but this does not conceptually make sense in a study like ours where both participants have the same role – all values of one participant could theoretically be swapped with the values of the other participant. We did initially try this left/right approach in an earlier stage of this study and achieved a maximum 51.0% accuracy, but accuracies for most approaches were slightly lower than in the current paper. In the end, we chose to take the mean and absolute difference of both participants’ values based on one previous study (Hernandez et al., 2014) that preliminarily used the value of one participant (the child in the adult-child dyad) and the difference between the participants’ values. However, there are many other ways that individual features from both participants could be combined, and no clear standard way to do it in the literature, so future studies may consider alternative approaches.

#### 4.2.2 Different experimental approaches

Even if physiological responses are not as useful as facial expressions or speech (used by our human raters to perform classification), intelligently combining them may still lead to higher overall accuracies than using either data source alone. In our study, preliminarily combining physiological responses with SAM and IIQ data only resulted in a small, statistically insignificant accuracy increase of 3%, but better results might be achieved with a more thorough attempt at combining data types. However, this would likely require the use of automated facial expression / speech analysis algorithms and would likely need to account for the fact that physiological processes operate on a different time scale than, e.g., facial expressions. For example, since EDA responses are often not visible for several seconds after an emotional event, a participant may already show and then hide a facial reaction to the event before the same event is reflected in the EDA signal. Similar timing mismatch issues are to some degree present between different physiological responses (with, e.g., heart rate reacting faster than skin temperature) and between physiological responses of both people (since one person may react faster than the other) but were considered beyond the scope of the current study since single-user studies predominantly use the same time scale for extraction of features from all physiological signals (Aranha et al., 2021; Novak et al., 2012) and since there is no standard way to handle timing offsets in dyads.

Additionally, in both the current study and our previous classification study (Chatterjee et al., 2023), both participants were aware of the desired conversation scenario (e.g., PV-HA) and followed the same instructions to induce it. This also meant that external observation and self-report ratings were relatively reliable and achieved high accuracies. However, external observation and self-report data may be less reliable if the two participants have different perceptions of the interaction. For example, in mental health counseling, a client may lose trust in their therapist but try not to show it, causing the counseling session to fail if the therapist does not notice and adjust their behavior. Similarly, in collaborative learning, a student may lose interest but try not to show it, leading to poor learning outcome if the teacher does not notice. In such situations, physiological responses may be relatively more useful due to the lower usefulness of other data sources. Studying this in a lab setting should be possible by, e.g., giving each participants different instructions in secret as preliminarily done in one of our other studies (Chatterjee et al., 2025).

Finally, applications of dyadic physiological responses in mental health counseling are likely to involve clinical populations – for example, individuals with depression or autistic individuals. In such cases, measurements such as facial expressions may again be less informative (since, e.g., autistic individuals are often considered less expressive by neurotypical observers), potentially increasing the usefulness of physiological measurements. It should be noted that some clinical populations (e.g., individuals with depression (Woody et al., 2016)) do exhibit different physiological responses to dyadic interaction compared to the general population; however, this does not necessarily mean that such physiological responses would not be useful.

## 5 Conclusion

We used classification algorithms to assign dyads’ autonomic nervous system responses to one of four conversation scenarios corresponding to positive/negative valence and high/low arousal. The highest obtained four-class classification accuracy was 51.5%, which is above chance level but below the accuracy achieved by human observers (69.6%) or participants’ self-report questionnaires (74.0%). The accuracy of physiology-based classification decreased to 43.1% if physiological synchrony features were removed. While these results should be considered exploratory due to the large number of tested algorithms, we tentatively conclude that dyadic autonomic nervous system responses can be used to classify the state of a conversation and that synchrony features are important for such estimation, but that dyadic physiological responses are not as accurate as human observers or self-report questionnaires.

As further research into the usefulness of dyadic physiological responses in affective computing, we propose both additional analyses as well as three new experimental directions: combining physiological responses with other data types, studying scenarios where participants have different impressions of the interpersonal interaction, and studying populations that would be relevant for mental health applications (e.g., people with depression). Such studies would provide further insight into the potential practical utility of dyadic physiological responses in education, mental health counseling and conflict resolution, where they could potentially improve outcomes if combined with information from other sources such as speech.

## Data Availability

The datasets presented in this study can be found in online repositories. The names of the repository/repositories and accession number(s) can be found: https://doi.org/10.5281/zenodo.13315682.
